# Stochastic analysis of Ebola infection in small zoonotic niches

**DOI:** 10.1098/rsos.240298

**Published:** 2024-11-13

**Authors:** Sena Mursel, Paolo Bocchini, Javier Buceta

**Affiliations:** ^1^ Department of Civil and Environmental Engineering, Lehigh University, Bethlehem, PA, USA; ^2^ Catastrophe Modeling Center, Lehigh University, Bethlehem, PA, USA; ^3^ Theoretical and Computational Systems Biology Program, Institute for Integrative Systems Biology (I2SyBio), CSIC-UV, Paterna, Spain

**Keywords:** stochastic SIR model, Ebola, zoonosis, mathematical epidemiology

## Abstract

The size of fruit bat colonies ranges from dozens to hundreds of thousands of individuals, depending on the species. While a deterministic modelling approach is appropriate for large colonies, the role of population fluctuations can be all-important for small colonies. From this perspective, we analyse the infection dynamics in small zoonotic niches due to filoviruses, e.g. Ebola. To this end, we perform stochastic numerical simulations and analytical calculations. The inherent stochasticity in ecological processes may play a significant role in driving small populations towards extinction. Here, we reveal that fluctuations can either lead to virus eradication or to sustain infection compared with the deterministic dynamics, depending on the size of the zoonotic niche. Altogether, our findings reveal non-trivial stochastic effects, which can shed light on the infection dynamics in small- and medium-sized bat colonies and help design preventive measures for zoonotic diseases.

## 1. Introduction

Infectious diseases exert a major burden on global public health and the global economy, especially in low-income countries [1,2]. In particular, diseases transmitted to humans from animal carriers, i.e. zoonoses, such as SARS, MERS, avian influenza, COVID-19 and Ebola virus disease (EVD), account for approximately 70% of the emerging infectious diseases [3]. In the case of EVD and other filovirus diseases (e.g. Marburg), fruit bats are believed to be one of the main reservoirs [4]. The first-ever recorded spillover of Ebola into humans occurred in Zaire (currently Democratic Republic of Congo) in 1976. Since then, and for more than four decades, a significant number of outbreaks have taken place in sub-Saharan Africa [5]. The 2013−2015 outbreak of EVD in West Africa has been the most severe outbreak so far and caused approximately 11 000 deaths [6]. Moreover, EVD is lethal among non-human primates like apes and monkeys, as well as other mammals, like antelopes, and during outbreaks a significant portion of the wildlife population is wiped out [7]. Thus, it is paramount to develop quantitative predictive tools to mitigate the devastating effects of zoonoses and, in particular, EVD.

Numerous models have been proposed in the context of infectious diseases to understand the conditions that lead to their propagation [8,9]. One particular framework that has been extensively used is compartmental models, where populations are divided into different subcategories depending on their status with respect to the disease, e.g. susceptible, infected, recovered and so on [10]. In that context, during the past years, several compartmental models have been proposed to understand the spreading of filoviruses from an ecological viewpoint, i.e. among the reservoir population [11–14]. However, these models usually consider that bat populations are ‘large’ and consequently the disease progression follows a deterministic behaviour. In other words, the common goal of these models is to capture the average dynamics and, for the law of large numbers, the overall behaviour of populations with a large number of individuals will be very similar to the average behaviour, which may be captured well by a deterministic model, especially if the problem is close to being linear.

The assumption of large enough colonies for the law of large numbers to apply is indeed well justified for some species of fruit bats (e.g. *Eidolon*) where the colonies comprise hundreds of thousands of individuals [15]. However, some species of fruit bats (e.g. *Hypsignathus monstrosus*) congregate in colonies that include as few as a dozen individuals [16]. In small colonies, the law of large numbers does not apply with the same force and the specific behaviour of each individual can significantly skew the whole population dynamics. For this reason, deterministic models are not appropriate, and stochastic models that can capture random fluctuations become necessary. As a matter of fact, the effect of internal fluctuations has been revealed to be of great significance in other zoonotic diseases, such as Hantavirus [17–22]. The size of what can be defined as a ‘small’ population of bats varies depending on the species and its ecological context. For example, the little brown bat roosts in hibernating colonies comprising up to $10^{5}$ individuals, although the average colony size is approximately 10^4^ [23,24]. Also, in the context of population recovery, a study mentioned approximately 10 individuals per cluster when almost approximately 10^3^ bats were present in the hibernaculum [25]. In this study, for the sake of establishing a clear and convenient categorization of population sizes, we define the reference sizes for ‘small’, ‘medium’ and ‘large’ populations as 10, 100 and 1000 individuals, respectively.

Herein, we propose a stochastic model of EVD propagation in bat colonies of different sizes that we analyse by means of numerical simulations and analytical approximations. By comparing the results with a deterministic version of the model, we conclude that fluctuations can lead to virus eradication for some trajectories, and therefore delay on average the onset of infection. Contrary to intuition, we also reveal that stochasticity can sustain infection with respect to the deterministic system depending on the size of the zoonotic niche. Finally, our article elaborates on the consequences of our results for designing preventive measures and preparedness against EVD and other zoonotic diseases.

## 2. Methods

### 2.1. Deterministic and stochastic SIR models

**Figure 1.**
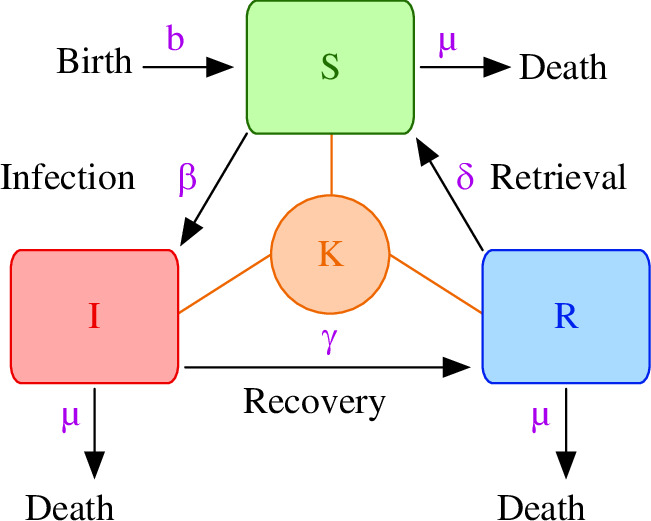
Schematic representation of the SIR model of EVD in a bat population. Bats are assumed to be born in a susceptible state ( S ) and after being infected ( I ) they can recover from the infection ( R ). Magenta letters indicated the nomenclature for the transition rates associated with the different transition events. Death rates, denoted as μ , are assumed to be the same regardless of the infection state. Competition for resources sets a carrying capacity K .


In this work, we follow the main ideas presented in the studies by Buceta and Johnson [13] and Fiorillo *et al.* [14] to model EVD epidemiology in bats’ zoonotic niches. In the model, the carrying capacity, K , represents the maximum number of individuals that the available resources at a given location can support. The model is schematically represented in figure 1. The different processes considered in the model among susceptible ( S ), infected ( I ), recovered ( R ) states can be represented in terms of the following transitions:


(2.1) $$\begin{array}{rlr} \text{Birth: }Z & \overset{b}{⟶} & Z+S, \end{array}$$

(2.2) $$\begin{array}{rlr} \text{Death: }Z & \overset{\mu }{⟶} & \emptyset , \end{array}$$

(2.3) $$\begin{array}{rlr} \text{Infection: }S+I & \overset{\beta }{⟶} & I+I, \end{array}$$

(2.4) $$\begin{array}{rlr} \text{Recovery: }I & \overset{\gamma }{⟶} & R, \end{array}$$

(2.5) $$\begin{array}{rlr} \text{Retrieval: }R & \overset{\delta }{⟶} & S, \end{array}$$

(2.6) $$\begin{array}{rlr} \text{Competition: }N+Z & \overset{\frac{\left|b-\mu \right|}{K}}{⟶} & N, \end{array}$$

where Z stands for S , I or R (i.e. the number of bats in compartment Z ) and N=S+I+R (i.e. the total number of individuals in compartments S , I and R ). The transition rates (i.e. the probability of events per unit of time), e.g. b , are assumed to be constant and need to be calibrated [14], as discussed later. Assuming that the total number of bats, N≫1 (i.e. a large population) a mass-action law approach leads to the deterministic SIR compartmental model [26,27] described in equations (2.7)–(2.9).


(2.7) $$\begin{array}{rl} \dot{S}\; & =\;bN-\mu S-\frac{\left|b-\mu \right|}{K}NS-\beta aSI\;+\delta R, \end{array}$$

(2.8) $$\begin{array}{rlr} \dot{I} & = & -\mu I-\frac{\left|b-\mu \right|}{K}NI+\beta SI-\gamma I, \end{array}$$

(2.9) $$\begin{array}{rlr} \dot{R} & = & -\mu R-\frac{\left|b-\mu \right|}{K}NR+\gamma I-\delta R. \end{array}$$

Consequently, from a deterministic perspective, the total population experiences logistic growth,


(2.10) $$\dot{N}=\left(b-\mu \right)N-\frac{\left|b-\mu \right|}{K}N^{2},$$

and at the steady state the population disappears if the birth rate is smaller than the death rate ( N=0 if b<μ ) or the population reaches the carrying capacity if the birth rate is larger than the death rate ( N=K if b>μ ). As previously shown (see [13,14]), in the deterministic model the infection is sustained in the population if $K>K_{c}=\frac{b+\gamma }{\beta }$ or, alternatively, if the basic reproduction number, $R_{0}=\beta K-b+\mu /\gamma +\mu =\beta /\gamma +\mu \left(K-K_{c}\right)+1>1$ [13,14]. That is, due to the logistic growth of the population, for a fixed set of parameter values, $R_{0}$ increases linearly with K .

When the population is small and fluctuations cannot be neglected, the set of transitions in equations (2.1)–(2.6) can be described by the following forward Kolmogorov equation (i.e. master equation) [28,29]:


(2.11) $$\begin{array}{rl} {\dot{P}}_{\left(S,I,R\right)} & =\;b\left(N-1\right)P_{\left(S-1,I,R\right)}+\left(S+1\right)\\ & \times \;\left(\mu +\frac{\left|b-\mu \right|}{K}N\right)P_{\left(S+1,I,R\right)}\\ & +\;\left(I+1\right)\left(\mu +\frac{\left|b-\mu \right|}{K}N\right)\\ & \times \;P_{\left(S,I+1,R\right)}+\left(R+1\right)\\ & \times \;\left(\mu +\frac{\left|b-\mu \right|}{K}N\right)P_{\left(S,I,R+1\right)}\\ & +\;\beta \left(S+1\right)\left(I-1\right)P_{\left(S+1,I-1,R\right)}\\ & +\;\gamma \left(I+1\right)P_{\left(S,I+1,R-1\right)}\\ & +\;\gamma \left(R+1\right)P_{\left(S-1,I,R+1\right)}\\ & -\;\{\left(b+\mu +\frac{\left|b-\mu \right|}{K}\left(N-1\right)\right)N+\beta SI\\ & +\;\gamma I+\delta R\}\;P_{\left(S,I,R\right)}, \end{array}$$

where $P_{\left(S,I,R\right)}$ is the probability of having S , I and R individuals at time t in the various compartments of the population.

As for the total population of bats, it is described by the transitions,


(2.12) $$\begin{array}{rlr} N & \overset{b}{⟶} & N+N, \end{array}$$

(2.13) $$\begin{array}{rlr} N & \overset{\mu }{⟶} & \emptyset , \end{array}$$

(2.14) $$\begin{array}{ccccc} & N+N & \overset{\frac{\left|b-\mu \right|}{K}}{⟶} & N & \end{array}$$

and the master equation for the probability of having N=S+I+R individuals at time t , $P_{N}$ , reads


(2.15) $$\begin{array}{rl} {\dot{P}}_{N} & =\;b\left(N-1\right)P_{N-1}+\left(N+1\right)\\ & \times \;\left(\mu +\frac{\left|b-\mu \right|}{K}N\right)P_{N+1}\\ & -\;\left(b+\mu +\frac{\left|b-\mu \right|}{K}\left(N-1\right)\right)N\;P_{N} \end{array}$$

### 2.2. Moment hierarchy breaking approach

There is no analytical solution for the master equations (2.11) and (2.15). However, the exact simulation of individual stochastic trajectories can be obtained by means of the Gillespie algorithm as discussed later. Unfortunately, the practical application of this approach sometimes leads to a high computational burden, because an accurate assessment of the probability distribution requires a very large number of samples to simulate.

Here, we implement the moment hierarchy breaking (MHB) approach, also referred to as the moment closure method, to obtain approximate analytical solutions for the first moments of the joint probability mass function $P_{\left(S,I,R\right)}$ and the probability mass function $P_{N}$ [30–32]. Our approach is based on the fact the cumulants, κ , are a way to characterize the probability distribution alternative to the calculation of its moments. Given a random variable X characterized by the moment generating function $M_{X}(t)$ , its cumulant-generating function $K_{X}(t)$ reads [33]


$$K_{X}(t)=log\left(M_{X}(t)\right)=log\langle e^{tX}\rangle .$$

Then, the cumulants $\kappa_{n}$ are derived from a power series expansion of $K_{X}(t)$ ,


$$\begin{array}{c} K_{X}(t)=\sum_{n=1}^{\infty }\kappa_{n}\frac{t^{n}}{n!}=\kappa_{1}\frac{t}{1!}+\kappa_{2}\frac{t^{2}}{2!}+\ldots \end{array}$$

Thus, the *n*th derivative of the previous expression evaluated at zero defines the *n*th-order cumulant,


$$\kappa_{n}=\frac{d^{n}K_{X}\left(t\right)}{dt^{n}}{|}_{t=0}.$$

Cumulants encapsulate the most significant statistical information of the respective random variables through their moments [34]. In fact, two probability distributions whose moments are identical will have identical cumulants and vice versa [35,36]. The first moments, $\mu_{i}$ and cumulants, $\kappa_{i}$ , satisfy the following relationships:


$$\begin{array}{rl} & \kappa_{1}\left(X\right)=\mu_{1}\left(X\right)=\langle X\rangle ,\\ & \kappa_{2}\left(X\right)=\mu_{2}\left(X\right)-\mu_{1}^{2}\left(X\right)=\langle X-\langle X\rangle \rangle^{2},\\ & \kappa_{3}\left(X\right)=\mu_{3}\left(X\right)-3\mu_{2}\left(X\right)\mu_{1}\left(X\right)+2\mu_{1}^{3}\left(X\right)=\langle X-\langle X\rangle \rangle^{3}, \end{array}$$

where ⟨⋅⟩ denotes the statistical average or expectation operator. Thus, in the particular case of a Gaussian distribution with mean μ and variance $\sigma^{2}$ the following holds:


$$\kappa_{1}\left(X\right)=\mu ,\;\kappa_{2}\left(X\right)=\sigma^{2},\;\kappa_{n}\left(X\right)=0,\;n\geq 3.$$

In the multivariate scenario, a similar rationale applies. Thus,


$$\begin{array}{rl} \kappa_{1}\left(X\right)=\kappa \left(X\right) & =\;\langle X\rangle ,\\ \kappa_{2}\left(X,Y\right)=\kappa \left(X,Y\right) & =\;\langle XY\rangle -\langle X\rangle \langle Y\rangle ,\\ \kappa_{3}\left(X,Y,Z\right)=\kappa \left(X,Y,Z\right) & =\;\langle XYZ\rangle -\langle XY\rangle \langle Z\rangle \\ & -\;\langle XZ\rangle \langle Y\rangle -\langle YZ\rangle \langle X\rangle \\ & +\;2\langle X\rangle \langle Y\rangle \langle Z\rangle , \end{array}$$

where in the context of this study, X , Y or Z stand for S , I or R and ⟨⋅⟩ denotes in this case the multivariate statistical average or expectation operator,


$$\langle f\left(S,I,R\right)\rangle =\sum_{S,I,R}f\left(S,I,R\right)P_{\left(S,I,R\right)}.$$

When considering the total population, N , the definition of the cumulants and the procedure explained in the following are straightforward because in this case X=Y=Z=N .

We then build an approximate solution by assuming that there exist the probability $P^{\text{approx}}$ for which the third-order cumulants are null, i.e. κ(X,Y,Z)=0 , and consequently third-order correlations can be approximated as


$$\begin{array}{rl} \left\langle XYZ\right\rangle & =\langle XY\rangle \langle Z\rangle +\langle XZ\rangle \langle Y\rangle \\ & +\langle YZ\rangle \langle X\rangle -2\langle X\rangle \langle Y\rangle \langle Z\rangle . \end{array}$$

This procedure, as shown in the appendix, allows us to break the hierarchy of moments and obtain closed-form ordinary differential equations for the moments ⟨X⟩ and ⟨XY⟩ . Consequently, the relationship κ(X,Y,Z)=0 implicitly assumes a Gaussian-like property for the probability $P^{\text{approx}}$ [34]. Notice that pushing the approximation further and imposing a moment closure relationship at the level of the second-order cumulants, i.e. κ(X,Y)=0 , would oversimplify the probabilistic description, lead to the deterministic solution, and consequently, the probability density would be approximated in that case by $P^{\text{approx}}=\delta \left(X-\langle X\rangle \right)\delta \left(Y-\langle Y\rangle \right)\delta \left(Z-\langle Z\rangle \right)$ . On the other hand, implementing the closure relationship at the level of the fourth-order cumulant makes the analytical calculations cumbersome and increases unnecessarily the complexity of the problem since, as shown below, the Gaussian approximation adequately captures the non-trivial stochastic effects. As a cautionary note, we stress that the Gaussian approximation, while accurate, is nonetheless unphysical since the described variables, i.e. populations, are implicitly allowed to take negative values. This fact can be particularly problematic when describing populations close to zero. Still, we note that when solving the MHB relationships for the statistical moments (see appendix), only non-negative solutions are allowed.

### 2.3. Numerical methods and parameters

Ordinary differential equations were solved using the fourth-order Runge–Kutta method as implemented in Matlab’s embedded library [37–39]. The simulations of the stochastic dynamics were performed using the Gillespie algorithm, in particular, using the direct method [40,41]. Numerical trajectories were simulated for various time windows spanning from one month to 50 years (see §3.3). As for the initial condition of our simulations, for every value of the carrying capacity, K , we explored $10^{3}$ random sets of initial conditions that satisfy the condition S+I+R=K with S , I and R greater than zero. We concluded that, as expected, as long as a large number of trajectories (i.e. stochastic realizations) are explored (see below) the ergodicity of the system ensures that a single initial condition is enough to capture the stationary probability density in full. On the other hand, when characterizing transient behaviours, averaging of different sets of initial conditions is key. In our case, to examine steady-state results, we typically used as initial condition 70% , 10% and 20% for susceptible, infectious and recovered states, respectively. Given that the maximum average lifespan of some bat species (e.g. *Rousettus aegyptiacus*) can be as long as 25 years (see parameter list below), the 50 -year simulation time frame is deemed sufficiently comprehensive to explore all relevant time scales in the model at the steady state [42,43]. To analyse statistical moments at the steady state, we computed averages over 10000 stochastic trajectories. The probability of sustained infections was defined as the ratio of trajectories resulting in infected bats at the end of the analysed time window to the total number of trajectories. In our simulations, we used the following parameter set, based on previous studies [14,43–45]. In particular, taking into account the range of variability described in [14], we used the mean values: b=1/365 day^−1^, μ=1/(27×365) day^−1^, γ=10/365 day^−1^, $\beta =3.9\times 10^{-4}$ day^−1^ and δ=11/365 day^−1^.

In our simulations, we also explored the effect of the duration of the observation window, T . This parameter dictates the duration of the simulation and it relates to how long it is reasonable to consider a colony as an isolated system. For example, through migration, the influx and efflux of individuals can change the epidemiological conditions of a colony. In this study, we considered the sensitivity of the results to the values of the observation window, T , ranging from one month (transient effects) to 50 years (steady state) using different sets of initial conditions as discussed above.

## 3. Results

### 3.1. Small populations are vulnerable to extinction due to stochasticity

In order to check the applicability of the MHB approach (see §2 and appendix), we first examined its results for the whole bat population, N=S+I+R , which should display a logistic growth behaviour, as shown in equation (2.10). Also in this case, the stochastic model is described by the transitions in equations (2.12)–(2.14) that define the master equation (2.15).

**Figure 2.**
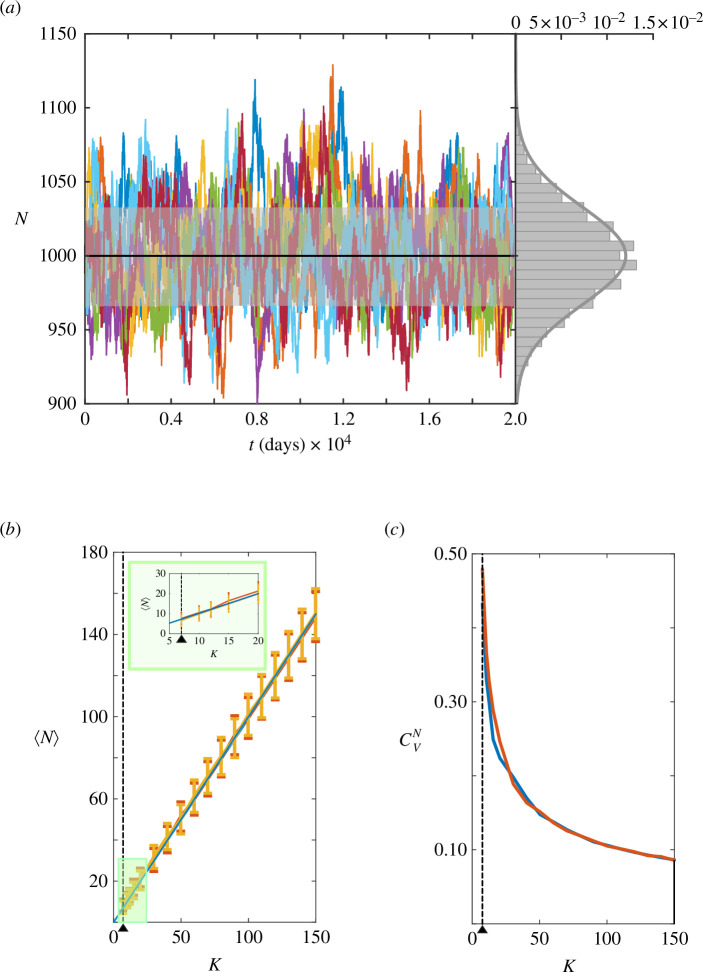
(*a*) The rugged colour lines show different stochastic trajectories for the total number of bats for K=1000 . The black line with the grey error band indicate the analytical calculation for $\langle N\rangle \pm \sigma_{N}=1000\pm 33$ . On the right, we show the probability density of N as obtained in numerical simulations (grey bars) and the Gaussian distribution derived from the MHB theory. (*b*) Comparison between numerical simulations (yellow), analytical calculations (orange) and the deterministic solution (blue) about the expected value of ⟨N⟩ at the stationary state as a function of K . Simulated time 20 years. The error band accounts for the standard deviation, $\sigma_{N}$ . (*c*) Coefficient of variation in numerical simulations (blue) and analytical calculations (orange) as a function of K . As K increases $c_{V}^{N}$ decreases and it is kept at low values thus precluding extinction events. The vertical dotted lines and the black triangles indicate the $K_{\text{lower}}$ value.

We performed stochastic simulations (see §2) for different values of the carrying capacity, K . For K , we only considered integer values because the total population of bats is the number of individuals. When K=1000≫1 , as expected, ${\left\langle N\right\rangle }_{K\gg 1}=K$ , i.e. as the carrying capacity increases, the average number of bats tends to the stationary deterministic solution: N=K . Figure 2*a* shows 10 stochastic trajectories as well as the analytical values for $\langle N\rangle \pm \sigma_{N}$ and the probability distribution obtained in the simulations and by the MHB approach.

An analysis of the 10000 trajectories reveals that the statistics for the first two moments obtained with the MHB method are indeed in excellent agreement with the analytical calculations for different values of K (table 1). In the table 1, the stochastic effects are quantified through the coefficient of variation (CoV), $c_{V}^{N}=\sigma_{N}/\left\langle N\right\rangle$ , that according to the analytical calculations (appendix) reads

**Table 1.** Comparison between analytical calculations and the values for ⟨N⟩ , $\sigma_{B}$ and $c_{V}^{N}$ obtained in numerical simulations for different values of K ( $10^{4}$ stochastic samples).

	analytical calculations			numerical simulations		
K	⟨N⟩	$\sigma_{N}$	$c_{V}^{N}$	⟨N⟩	$\sigma_{N}$	$c_{V}^{N}$
7	6.60	3.04	0.46	7.55	2.90	0.38
10	9.79	3.44	0.35	11.80	3.44	0.29
12	11.83	3.72	0.31	12.45	3.75	0.30
50	49.94	7.28	0.15	51.51	7.19	0.14
100	99.95	10.24	0.10	101.45	10.18	0.10
500	499.95	22.81	0.045	501.61	22.66	0.045
1000	999.96	32.24	0.032	1001.80	31.96	0.032
5000	5000.00	72.06	0.014	5000	71.98	0.014


(3.1) $$c_{V}^{N}=\sqrt{\frac{\left(1+K-\sqrt{1+K\left(2-\frac{8b}{b-\mu }+K\right)}\right)}{\left(3+3K+\sqrt{1+K\left(2-\frac{8b}{b-\mu }+K\right)}\right)}}.$$

Thus, $\underset{K\to \infty }{\;\lim\;}c_{V}^{N}=0$ and, as expected, the stochastic effects are negligible for large populations. As shown in equation (3.1), $c_{V}^{N}$ incorporates square roots and we emphasize that $c_{V}^{N}$ needs to be a real and positive value by definition. Furthermore, K can only take integer values. Thus, equation (3.1) is explicitly defined for K values greater than or equal to a predetermined lower bound, $K_{\text{lower}}$ . This lower limit, $K_{\text{lower}}$ , is determined as $\left\lceil \frac{3b+\mu +\sqrt{8b\left(b+\mu \right)}}{b-\mu }\right\rceil$ where ⌈⋅⌉ represents the ceiling function. For instance, in our simulations and the specified values of b and μ (§2), the lower bound for K becomes 7 . This indicates that the expression yields a complex value when K is less than 7 . For the same values of b and μ in our simulations, $c_{V}^{N}$ can approach values near 0.5 for K close to $K_{\text{lower}}$ , and the trend for $c_{V}^{N}$ values consistently decreases as K increases. Consequently, for K values smaller than $K_{\text{lower}}$ , even without an analytical expression, the observed trend suggests an increase in the coefficient of variation. A coefficient of variation of more than 50% for small populations is very substantial and these results, indeed, indicate that stochasticity has a notable effect over the whole population and that fluctuations can lead to systematic extinction events in small niches. As an illustration, when K is set to 12, representing a reasonable colony size for *H. monstrosus*, the CoV is calculated to be 31.4% [46]. In fact, based on our simulations, the observed CoV in this specific case is 30.2% (see table 1). To evaluate the agreement between the analytical approximations and numerical results, we also ran simulations in small- to medium-sized niches: K∈(7,150) , figure 2*b*. The results reveal that (i) ⟨N⟩ follows the deterministic behaviour, (ii) the MHB approach and the simulations are in agreement, and (iii) the probability of extinction becomes essential for small populations.

### 3.2. Fluctuations result in a higher probability of virus eradication in zoonotic niches

From a deterministic viewpoint, an analysis of the transition towards the infective state indicates the existence of a threshold value of the carrying capacity, $K_{c}$ (or equivalently as a function of the basic reproduction number $R_{0}$ , see §2) [13]. If $K<K_{c}=\left(b+\gamma \right)/\beta$ , i.e. if $R_{0}<1$ , (where β is the infection rate) at steady state the population is free of infection and S=K . On the other hand, if the size of the zoonotic niche, K , is larger than $K_{c}$ , i.e. if $R_{0}>1$ , the infection propagates and $S=\frac{b+\gamma }{\beta }$ , $I=\frac{b+\delta }{b+\gamma +\delta }\left(K-K_{c}\right)$ and $R=\frac{\gamma }{b+\gamma +\delta }\left(K-K_{c}\right)$ . Notice that regardless of the infective state (i.e. free of infection or not) at steady state N=S+I+R=K .

**Figure 3.**
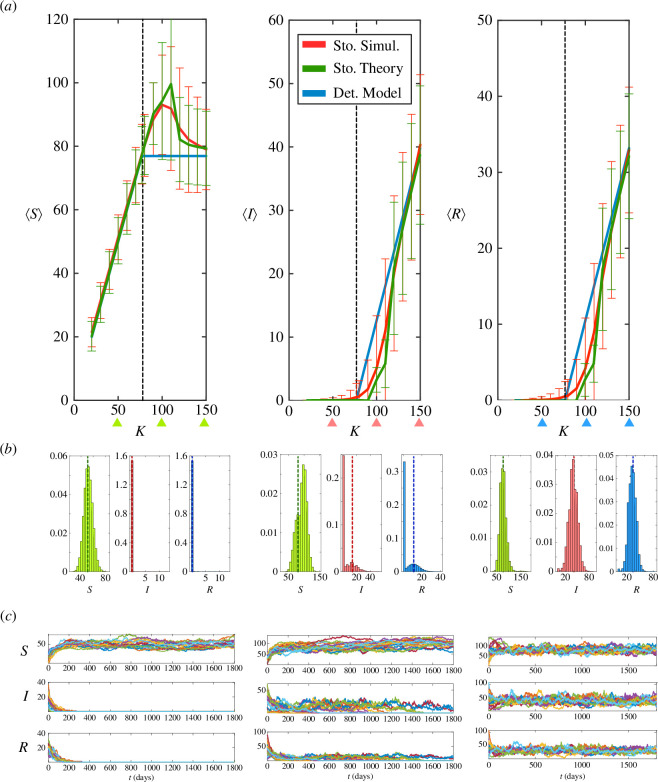
(*a*) From left to right, the panels show the stationary solution (solid lines) of the average susceptible ⟨S⟩ , infected ⟨I⟩ and recovered ⟨R⟩ bats as a function of the carrying capacity (i.e. size of the zoonotic niche), K , as predicted by stochastic simulations (red curve), the MHB theoretical calculations (green curve) and the deterministic model (blue curve). The goodness of the MHB theory is also noticeable by the estimation of the second moments as reflected by the errors bars (standard deviation). The vertical black dashed line indicates the critical value of the carrying capacity, $K_{c}≃77$ , as predicted by the deterministic model. Parameter values as indicated in the main text. In the stochastic simulations $10^{3}$ realizations were considered (simulation time 50 years). (*b*) Normalized histograms (i.e. probability density) of susceptible, infected and recovered bats as obtained in the stochastic simulations for K=50 (left), K=100 (centre) and K=150 (right) (triangles in panel (*a*)). The vertical dotted lines indicate the expected deterministic value. (*c*) Stochastic trajectories ( n=20 , randomly selected) of S , I and R as a function of time and starting from different initial conditions. From left to right the values of K as indicated in panel (*b*).

In order to evaluate the effect of the population fluctuations that originate from a stochastic description, we first analyse the results of the MHB approach (see §2). Interestingly, the analytical calculations predict that the average population of susceptible bats ⟨S⟩ increases as K for values of K slightly larger than $K_{c}$ , $K≳K_{c}$ , as seen in figure 3*a*. Consequently, since the total average bat population in the steady state is conserved also in the stochastic model (i.e. ⟨N⟩=⟨S+I+R⟩=K ), it can be concluded that the fluctuations delay, on average, the appearance of infection compared with the deterministic model. Such a delay can also be observed by studying the correlations between S , I and R (appendix, figure 6). Also, as expected, when K is higher, the stochastic solution approaches the deterministic solution, $\langle S\rangle ≃\left(b+\gamma \right)/\beta =K_{c}≃77$ . As a result, the infection-buffering effect induces a non-monotonic behaviour of ⟨S⟩ . Finally, we performed stochastic simulations to validate the aforementioned theoretical results, figure 3*a*. The simulations confirm the MHB theoretical predictions qualitatively and quantitatively and we can conclude that the fluctuations of the bat population lead to a non-null probability of eradication of the Ebola infection in medium size populations.

In order to shed light on the observed behaviour, we computed the probability density of susceptible, infected and recovered bats for values of the carrying capacity lower, similar to and larger than the threshold $K_{c}$ (figure 3*b*). The average values of S , I and R approach their deterministic values when K is either significantly smaller or larger than $K_{c}$ . However, for K similar to $K_{c}$ the probability density reveals the discrepancies shown in figure 3*a* and the reason underlying such a mismatch. Given the low numbers of infected and recovered bats, near $K_{c}$ the fluctuations of the population drive their extinction and the population splits in two categories. Survival trajectories of I and R satisfy, on average, the deterministic behaviour. However, a number of their trajectories become extinct and consequently drive the average of the probability density towards lower values. Since I=R=0 is an absorbing state and the total average numbers of bats is conserved, the amount of susceptible bats necessarily increases when the infection is eradicated. As for the possible effect of the initial condition, figure 3*c* shows stochastic trajectories for the values of K analysed in figure 3*b* ( 50 , 100 and 150 ). These trajectories were generated using random initial conditions (but ensuring (S,I,R)>0 and S+I+R=K , see §2). As expected, the initial condition is irrelevant at the steady state since the system is ergodic. Thus, although the initial conditions result in varying trajectories, they all ‘converge’ to the anticipated behaviour given the value of K .

### 3.3. The probability of sustained infections reveals stochastic effects as a function of the observation window

Given the above results, we explored deeper how infection in a colony is modulated by fluctuations. To that end, we computed the probability of sustained infections, $P_{\text{Inf.}}$ . Namely, the probability that in a stochastic trajectory of duration T (i.e. an observation window of duration T ), I≠0 at time T (see §2).

**Figure 4.**
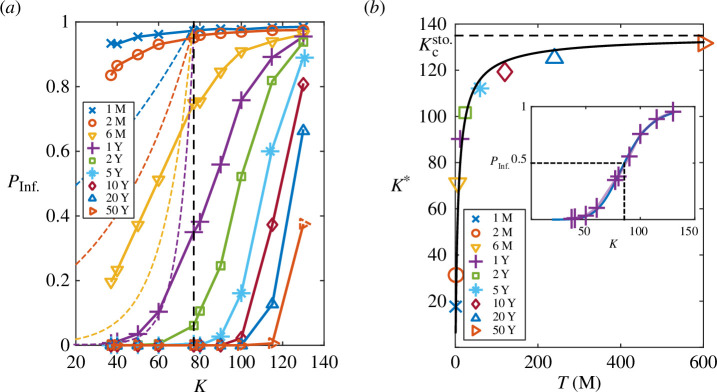
(*a*) Probability of sustained infections, $P_{\text{Inf.}}$ , as a function of K and different values of T (in the legend, M and Y stand for months and years, respectively). Solid lines with symbols represent stochastic simulations (based on $10^{4}$ stochastic realizations while dashed lines show deterministic estimations. The dashed vertical black line indicates the population threshold (i.e. critical size of the zoonotic niche) to sustain the infection in the deterministic system: $K_{c}≃77$ . (*b*) Estimation of the population threshold to sustain the infection in the stochastic system (see text), $K_{c}^{\text{sto.}}=135$ . The inset shows an example of sigmoid fitting of $P_{\text{Inf.}}$ for the case T=1 year, where $K^{*}≃90$ is determined by the value of K at which $P_{\text{Inf.}}=1/2$ .

Figure 4*a* shows $P_{\text{Inf.}}$ for different values of T . On the one hand, the results reveal, in agreement with figure 3, that if $K>K_{c}$ the fluctuations lead to an increased probability of virus eradication. Namely, if $K>K_{c}$ , $P_{\text{Inf.}}=1$ in the deterministic system, but in the stochastic approach the infection is reduced and $P_{\text{Inf.}}<1$ . Also, as the observation window, T , increases the stochastic effects become more evident because larger values of the zoonotic niche, K , are needed for the infection to survive with respect to the deterministic behaviour for which $P_{\text{Inf.}}=1$ if $K>K_{c}$ independently of the value of T .

Figure 4*a* also suggests that in the limit for T→∞ (i.e. at the steady state) there exist a stochastic critical threshold of the carrying capacity, $K_{c}^{\text{sto.}}$ , below which the infection dies out. To test this hypothesis, we determined the stochastic transition points, $K^{*}$ (symbols in figure 4*b*), as a function of T by fitting the curves $P_{\text{Inf.}}(K)$ to a Hill sigmoid function for each investigated value of T (equation (3.2)).


(3.2) $$P_{\text{Inf.}}\left(K\right)=\frac{K^{n}}{{\left(K^{*}\right)}^{n}+K^{n}}.$$

As expected, figure 4*b* reveals that, as T increases, the value of $K^{*}$ increases and approaches an asymptote that defines the stochastic critical threshold of the carrying capacity, $K_{c}^{\text{sto.}}$ . In order to determine $K_{c}^{\text{sto.}}$ , we fitted our data to the exponential behaviour given in equation (3.3),


(3.3) $$\begin{array}{ccc} & K^{*}\left(T\right)=K_{c}^{\text{sto.}}{\left(1-e^{-\epsilon T^{\zeta }}\right)}^{\eta } & \end{array}$$

and found that the best fit is given by values ϵ=13.94 , ζ=0.0475 , $\eta =3.5\times 10^{6}$ and $K_{c}^{\text{sto.}}=135$ (black solid line in figure 4*b*). Thus, our data indicate that, at the steady state, the stochasticity increases the size of the zonootic niche required to sustain infection by approximately 75% with respect to the deterministic value. We also note that $K_{c}^{\text{sto.}}$ determines the value of the carrying capacity at which the stochastic and deterministic solutions for the means, ⟨S⟩ , ⟨I⟩ and ⟨R⟩ , begin converging.

On the other hand, we observed that if $K<K_{c}$ the fluctuations, depending on the value of T and K , may either help to sustain or to eradicate the infection with respect to the deterministic behaviour (figure 4*a*). In fact, the probability to sustain infections at a time T in the deterministic system can be estimated as (see details in appendix)


(3.4) $$P_{\text{Inf.}}^{\text{det.}}≃\theta \left(K-K_{c}\right)+\theta \left(K_{c}-K\right)e^{-T\cdot \beta \left(K_{c}-K\right)},$$

where θ(⋅) is the Heaviside step function. When compared with the data from stochastic simulations, the results suggest that when T is small enough (e.g. T= one month) the stochasticity sustains infection. That is $P_{\text{Inf.}}^{\text{sto.}}>P_{\text{Inf.}}^{\text{det.}}$ . However, as the observation window, T , increases (e.g. T=1 year) the fluctuations contribute to mitigate the infection as when $K>K_{c}$ . We notice, however, that the deterministic estimate, equation (3.4), is based on a linear approximation, and consequently, a precise quantitative comparison is not possible (§4).

### 3.4. The extinction time for infection diverges as the carrying capacity approaches the stochastic critical threshold

**Figure 5.**
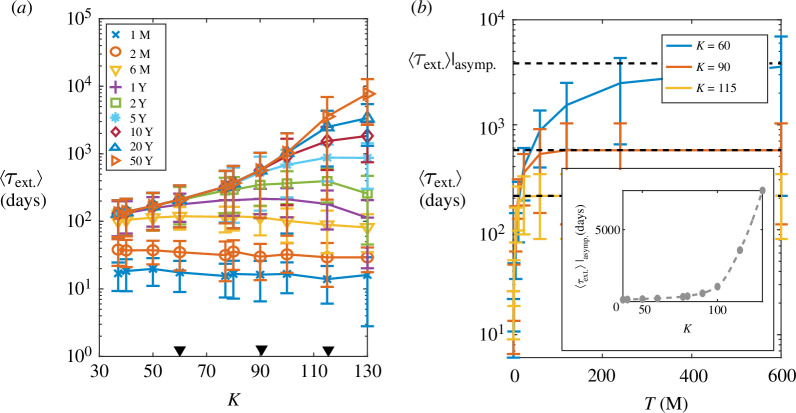
(*a*) Average time for infection extinction, $\left\langle \tau_{\text{ext.}}\right\rangle$ , as a function of the size of the zoonotic niche, K , for different observation window values, T (where M and Y stand for months and years respectively; $10^{4}$ stochastic realizations). Error bars: standard deviation. (*b*) $\left\langle \tau_{\text{ext.}}\right\rangle$ as a function of T for some values of K (represented by black solid triangles in panel (*a*)). Symbols denote stochastic simulations (error bars: standard deviation), solid lines indicate exponential fitting (see text) and dashed lines represent values of $\langle \tau_{\text{ext.}}\rangle {|}_{\text{asymp.}}$ as determined by the fitting. The inset shows $\langle \tau_{\text{ext.}}\rangle {|}_{\text{asymp.}}$ as a function of K .


**Figure 6.**
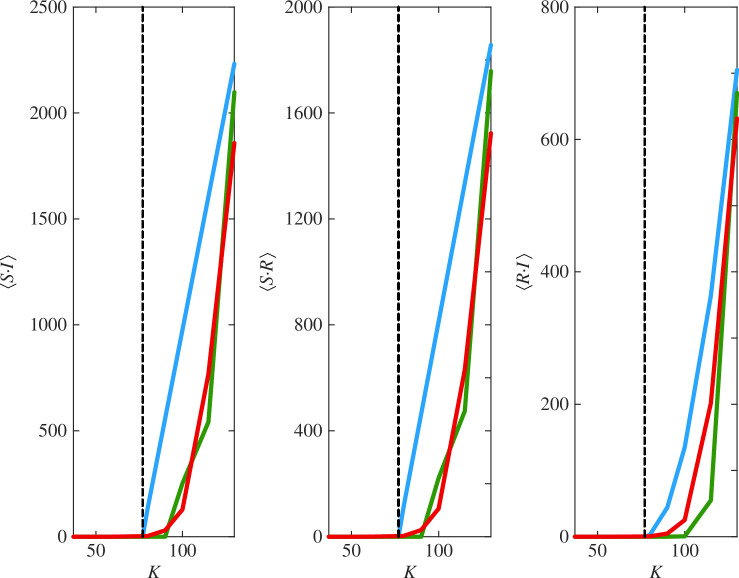
Correlations among species in the deterministic (blue line) and stochastic models (red line: numerical simulations; green line: MHB theory). The vertical dotted line indicates the transition points towards infection for the deterministic model ( $K_{c}≃77$ ).

To further assess the dynamics of infection extinction depending on the size of the zoonotic niche, K , we estimated the mean time for the infection to die $\left\langle \tau_{\text{ext.}}\right\rangle$ , figure 5*a*. To that end, we use different observation windows of duration T . In other words, given a particular observation window, T , and a size of the zoonotic niche, K , we evaluated the average time it takes for a trajectory to reach I=0 . Thus, for a given value of T , $\left\langle \tau_{\text{ext.}}\right\rangle$ is calculated considering only trajectories where the number of infected bats reaches zero at times t<T . In this regard, we point out that if the parameters are such that the infection becomes extinct $\left\langle \tau_{\text{ext.}}\right\rangle$ is independent of T . However, in practical terms, this requires to run long simulations to estimate $\left\langle \tau_{\text{ext.}}\right\rangle$ accurately. To overcome this issue, we implement an approach that allows us to extrapolate the asymptotic behaviour from simulations by using different values of T . Our results indicate that if $K<K_{c}^{\text{sto.}}$ , there is a convergence as T increases (i.e. the asymptotic behaviour of $\left\langle \tau_{\text{ext.}}\right\rangle$ is well defined). However, as K approaches $K_{c}^{\text{sto.}}$ convergence requires larger values of T . This observation, in agreement with figure 4*b*, suggests that infection is sustained if $K>K_{c}^{\text{sto.}}$ . These results are more clearly shown by plotting $\left\langle \tau_{\text{ext.}}\right\rangle$ as a function of T for different values of K , figure 5*b*. In that panel, to determine the asymptotic value of $\left\langle \tau_{\text{ext.}}\right\rangle$ , we proceed as in the case of $K^{*}$ (figure 4*b*) by fitting the curves to an exponential function (equation (3.5)),

(3.5) $$\langle \tau_{\text{ext.}}\rangle \left(T\right)=\langle \tau_{\text{ext.}}\rangle {|}_{\text{asymp.}}{\left(1-e^{-\theta T^{\lambda }}\right)}^{\omega }.$$

By using the asymptotic fitted values, as shown in the inset in figure 5*b*, we observed that $\langle \tau_{\text{ext.}}\rangle {|}_{\text{asymp.}}$ increases as K increases and shows a diverging behaviour as the size of the zoonotic niche approaches $K_{c}^{\text{sto.}}$ . These results highlight that depending on the colony size, i.e. on the value of K , an accurate estimation of the average time for an infection to die out requires very different observation times. Importantly, this fact should be considered in relation to the amount of time that the zoonotic niche can be consider isolated (§4).

Everything considered, from both the viewpoints of the probability of sustained infections and the extinction time, our results indicate that the fluctuations of the population act as a protective mechanism against infection such that the critical size of the zoonotic niche for which infection is sustained increases with respect to the expected deterministic behaviour.

## 4. Conclusions and discussion

The global challenge of epidemic disease transmission has sought the attention of decision-makers, as it poses significant threats to both human lives and economic stability. Researchers are driven by the urge to comprehend this issue and propose strategies to mitigate the spread of such diseases. The first generation of models emerged in the form of deterministic frameworks. However, these models exhibited certain limitations in their realism due to their disregard for the influence of intrinsic and extrinsic fluctuations. Recent advancements in the field have led to a more profound understanding of disease outbreaks by incorporating the intrinsic randomness inherent in such processes [47,48]. Herein, through rigorous theoretical analysis using an MHB approach, we present closed-form solutions that account for stochastic effects. Numerical results provide evidence that the outcomes derived from the theoretical framework align closely with the results obtained through numerical analysis. In particular, our results reveal that stochasticity leads to a non-null probability to eradicate infections, and as a result the size of the zoonotic niches that sustain infection is larger than the deterministic expectation. Moreover, our calculations also suggest that under some conditions, the fluctuations are able to sustain the infection for longer times than in a deterministic system. Nonetheless, in this case, a cautionary tale is needed when comparing the deterministic and stochastic models. By definition, the probability of infection in the deterministic model is either 1 or 0 . To circumvent this issue and estimate a continuous value of the probability, i.e. $P_{\text{Inf.}}^{\text{det.}}\in \left(0,1\right)$ , we implemented a linear approach (appendix). Consequently, the comparison between the deterministic (linear) model and the stochastic (nonlinear) model must be carefully analysed.

It is also important to acknowledge the limitations of the MHB technique. The multivariate Gaussian approximation implemented through the MHB method explains very well the values of the two first moments obtained in the simulations but disregards correlations among variables beyond the second order. This introduces a level of approximation and is one of the reasons why there is some disparity between analytical and numerical results (another one being the numerical errors and the finite statistics of our simulations). Related to this, in our study, we have broken the moments’ hierarchy at the third order, but we could have extended this approach to higher orders by implementing, for example, that the fourth-order cumulant is null. In that case, the equations to be solved increase from 9 to 19 and the analytical calculations become cumbersome; but we could expect even better results. However, given the excellent agreement, we have obtained in our study we have deemed this to be unnecessary. Altogether, while quantitative predictions from the theoretical analysis could be certainly improved, the stochastic effects that we report here will still be present and the MHB approach when assuming ‘Gaussianity’ capture them convincingly well.

Our modelling approach also includes some important assumptions. Namely, the independence of the carrying capacity from environmental factors and, consequently, the implicit assumption that those are constant, despite their time-dependent nature. Additionally, parameters such as the birth rate often exhibit temporal variations. These assumptions stress the importance of the analyses carried out as a function of the observation window, T . This quantity informs about the amount of time that one could consider the zoonotic niche to be isolated (lack of influx/efflux of individuals) and subjected to invariable conditions. We have shown that, as expected, transient behaviours may differ significantly from the steady-state conditions (figures 4 and 5). The analysis of the infection probability in relation to the carrying capacity and the duration of the simulation time frame demonstrates a multi-faceted interplay between various variables that impact the dynamics of the disease. Particularly, in short-term simulations (e.g. one month), it appears that the solutions generated do not guarantee a steady-state outcome. As a result, the reliability of these short-term simulations tends to be comparatively lower. Nevertheless, these outcomes still hold important value, especially when devising strategies for short-term intervention mechanisms. Conversely, in longer time frames, in both deterministic and stochastic cases, the critical values dictating the onset of infection exhibit a significant discrepancy. This phenomenon diverges from the initial expectation that these critical values would gradually converge as the simulation duration extends. The observed divergence is not unique to this particular study but is a phenomenon that surfaces in the context of various diseases, including instances like Hantavirus modelling where stochasticity plays a role [17].

Our conclusions have direct implications. Vector control actions must continue not only when the critical values are attained but also for values nearby such values taking into account the potential presence of intrinsic fluctuations, especially when small and medium-sized niches are encountered. Standard procedures involve using $R_{0}$ , the basic reproduction number, to consider potential control and mitigation measures. That is, if the number is greater than 1, action needs to be taken to prevent the spread of the disease. This study, however, demonstrates that this approach alone might be insufficient. Stochastic analysis reveals the reverse of what the deterministic model, which corresponds to a single value of infection point, predicts. The theoretical framework shows that the potential variation due to bat dynamics might directly lead to a spark to start an epidemic, thus control measures must be integrated with stochasticity [49]. This study can help future Ebola epidemic preparedness and contingency plans, particularly in small and medium-sized zoonotic niches [50]. In particular, increased wildlife disease surveillance is required, and data from this surveillance must be incorporated into model development and analysis to hinder spillover [51,52].

Our framework has been applied in this study to address the EVD, but its applicability can extend to other zoonotic diseases, including avian influenza, Marburg and Zika viruses. While some parameters and the SIR model may vary, the theory still remains valid, as long as the infection dynamics are driven by the population density. Any necessary modifications can be readily implemented, enabling the calculation of the infected animal population across varying carrying capacities. Effects of intrinsic fluctuations are a function of different parameters leading to a spectrum of effects ranging from minimal to substantial. For example, for Marburg virus, if the model yields similar patterns as observed in the Ebola virus model presented in this manuscript, including zoonotic niches in the analysis can identify locations that may be more prone to disease spillover [53].

Altogether, herein we have shown important, non-trivial, stochastic effects that can hopefully help to understand the infection dynamics of zoonotic niches.

## Data Availability

The manuscript contains all the data, along with corresponding appendixes and electronic supplementary material. Access to the scripts/codes is available through the Figshare repository [54].

## Appendix

### Master equation: cumulants and moment hierarchy breaking approach

The following equation describes the master equation for entire bat population, N=S+I+R ,

$$\frac{\partial P_{N}}{\partial t}=b\left(N-1\right)P_{N-1}+\left(N+1\right)\left(\mu +\frac{\left|b-\mu \right|}{K}N\right)P_{N+1}-\left(b+\mu +\frac{\left|b-\mu \right|}{K}\left(N-1\right)\right)NP_{N}.$$

The n -moment of the $P_{N}$ distribution reads $\langle N^{n}\rangle =\sum_{N=1}^{\infty }N^{n}P_{N}$ , and by manipulating the previous equation, we obtain the following dynamical equations for the first two moments:

$$\begin{array}{rl} \langle \frac{\partial N}{\partial t}\rangle = & \langle N\rangle (b-\mu )\left(1+\frac{1}{K}\right)-\langle N^{2}\rangle \left(\frac{b-\mu }{K}\right),\\ \langle \frac{\partial N^{2}}{\partial t}\rangle = & -2\langle N^{3}\rangle \left(\frac{b-\mu }{K}\right)+\langle N^{2}\rangle (b-\mu )\left(2+\frac{3}{K}\right)+\langle N\rangle \left(b+\mu -\frac{b-\mu }{K}\right). \end{array}$$

By imposing the closure condition (see text) $\kappa_{3}\left(N\right)=\langle N^{3}\rangle -3\langle N^{2}\rangle \langle N\rangle +2\langle N\rangle^{3}=0$ , we obtain the following stationary solutions for ⟨N⟩ and $\left\langle N^{2}\right\rangle$ :

$$\begin{array}{rl} \left\langle N\right\rangle & =\frac{1}{4}\left(3+3K+\sqrt{1+K\left(2-\frac{8b}{b-\mu }+K\right)}\right), \end{array}$$

$$\begin{array}{ll} \left\langle N^{2}\right\rangle & =\frac{1}{4}\left(K+1\right)\cdot \left(3+3K+\sqrt{1+K\left(2-\frac{8b}{b-\mu }+K\right)}\right). \end{array}$$

As for the case where the whole population is split in terms of S , I and R , the master equation reads

$$\begin{array}{rl} \frac{\partial P_{\left(S,I,R\right)}}{\partial t} & =b\left(S+I+R-1\right)P_{\left(S-1,I,R\right)}\\ & \;+\left(\mu \left(S+1\right)+\frac{|b-\mu |}{K}\left(S+I+R\right)\left(S+1\right)\right)P_{\left(S+1,I,R\right)}\\ & \;+\left(\mu \left(I+1\right)+\frac{|b-\mu |}{K}\left(S+I+R\right)\left(I+1\right)\right)P_{\left(S,I+1,R\right)}\\ & \;+\left(\mu \left(R+1\right)+\frac{|b-\mu |}{K}\left(S+I+R\right)\left(R+1\right)\right)P_{\left(S,I,R+1\right)}\\ & \;+\beta \left(S+1\right)\left(I-1\right)P_{\left(S+1,I-1,R\right)}+\gamma \left(I+1\right)P_{\left(S,I+1,R-1\right)}\\ & \;+\delta \left(R+1\right)P_{\left(S-1,I,R+1\right)}\\ & \;-b\left(S+I+R\right)P_{\left(S,I,R\right)}-\mu \left(S+I+R\right)P_{\left(S,I,R\right)}\\ & \;-\frac{|b-\mu |}{K}\left(S+I+R\right)\left(S+I+R-1\right)P_{\left(S,I,R\right)}\\ & \;-\beta SIP_{\left(S,I,R\right)}-\gamma IP_{\left(S,I,R\right)}-\delta RP_{\left(S,I,R\right)}. \end{array}$$

Thus, the dynamical equations for the first two moments of the different species become:

$$\begin{array}{rl} \left\langle \frac{\partial S}{\partial t}\right\rangle & =b\langle S+I+R\rangle -\mu \langle S\rangle -\frac{|b-\mu |}{K}\langle S\cdot (S+I+R-1)\rangle -\beta \langle S\cdot I\rangle +\delta \langle R\rangle \\ \left\langle \frac{\partial I}{\partial t}\right\rangle & =-\mu \langle I\rangle -\frac{|b-\mu |}{K}\langle I\cdot (S+I+R-1)\rangle +\beta \langle S\cdot I\rangle -\gamma \langle I\rangle \\ \left\langle \frac{\partial R}{\partial t}\right\rangle & =-\mu \langle R\rangle -\frac{|b-\mu |}{K}\langle R\cdot (S+I+R-1)\rangle +\gamma \langle I\rangle -\delta \langle R\rangle \\ \left\langle \frac{\partial S^{2}}{\partial t}\right\rangle & =b\langle S+I+R\cdot (2S+1)\rangle -\mu \langle S\cdot (2S-1)\rangle -\frac{|b-\mu |}{K}\langle S\cdot (S+I+R-1)\cdot \\ & \cdot (2S-1)\rangle -\beta \langle S\cdot I\cdot (2S-1)\rangle +\delta \langle R\cdot (2S+1)\rangle \\ \left\langle \frac{\partial I^{2}}{\partial t}\right\rangle & =-\mu \langle I\cdot (2I-1)\rangle -\frac{|b_{K}-\mu |}{K}\langle I\cdot (2I-1)\cdot (S+I+R-1)\rangle \\ & +\beta \langle S\cdot I\cdot (2I+1)\rangle -\gamma \langle I(2I-1)\rangle \\ \left\langle \frac{\partial R^{2}}{\partial t}\right\rangle & =-\mu \langle R\cdot (2R-1)\rangle -\frac{|b-\mu |}{K}\langle R\cdot (S+I+R-1)\cdot (2R-1)\rangle \\ & +\gamma \langle I\cdot I\cdot (2R+1)\rangle -\delta \langle R\cdot (2R-1)\rangle \end{array}$$

and the cross terms of species S , I and R read:

$$\begin{array}{rl} \left\langle \frac{\partial SI}{\partial t}\right\rangle & =b\langle I\cdot (S+I+R)\rangle -2\mu \langle S\cdot I\rangle -2\frac{|b-\mu |}{K}\langle S\cdot I\cdot (S+I+R-1)\rangle \\ & +\beta \langle S\cdot I\cdot (S-I-1)\rangle +\delta \langle I\cdot R\rangle \\ \left\langle \frac{\partial SR}{\partial t}\right\rangle & =b\langle R\cdot (S+I+R)\rangle -2\mu \langle S\cdot R\rangle -2\frac{|b-\mu |}{K}\langle S\cdot R\cdot (S+I+R-1)\rangle \\ & +\beta \langle S\cdot I^{2}\rangle +\gamma \langle S\cdot I\rangle +\delta \langle R\cdot (R-S-1)\rangle \\ \left\langle \frac{\partial IR}{\partial t}\right\rangle & =-2\mu \langle I\cdot R\rangle -2\frac{|b-\mu |}{K}\langle I\cdot R\cdot (S+I+R-1)\rangle +\beta \langle S\cdot I\cdot R\rangle \\ & +\gamma \langle I\cdot (I-R-1)\rangle -\delta \langle I\cdot R\rangle \end{array}$$

On the other hand, the following equations describe the third-order cumulants of the multivariate probability functions $P_{\left(S,I,R\right)}$ :

$$\begin{array}{rl} \kappa (S,I,R) & =\langle S\cdot I\cdot R\rangle -\langle S\cdot I\rangle \cdot \langle R\rangle -\langle S\cdot R\rangle \cdot \langle I\rangle -\langle I\cdot R\rangle \cdot \langle S\rangle +2\langle S\rangle \cdot \langle I\rangle \cdot \langle R\rangle \\ \kappa (S,S,I) & =\langle S^{2}\cdot I\rangle -\langle S^{2}\rangle \cdot \langle I\rangle -2\langle S\cdot I\rangle \cdot \langle S\rangle +2\langle S\rangle^{2}\cdot \langle I\rangle \\ \kappa (S,S,R) & =\langle S^{2}\cdot R\rangle -\langle S^{2}\rangle \cdot \langle R\rangle -2\langle S\cdot R\rangle \cdot \langle S\rangle +2\langle S\rangle^{2}\cdot \langle R\rangle \\ \kappa (I,I,S) & =\langle I^{2}\cdot S\rangle -\langle I^{2}\rangle \cdot \langle S\rangle -2\langle I\cdot S\rangle \cdot \langle I\rangle +2\langle I\rangle^{2}\cdot \langle S\rangle \\ \kappa (I,I,R) & =\langle I^{2}\cdot R\rangle -\langle I^{2}\rangle \cdot \langle R\rangle -2\langle I\cdot R\rangle \cdot \langle I\rangle +2\langle I\rangle^{2}\cdot \langle R\rangle \\ \kappa (R,R,S) & =\langle R^{2}\cdot S\rangle -\langle R^{2}\rangle \cdot \langle S\rangle -2\langle R\cdot S\rangle \cdot \langle R\rangle +2\langle R\rangle^{2}\cdot \langle S\rangle \\ \kappa (R,R,I) & =\langle R^{2}\cdot I\rangle -\langle R^{2}\rangle \cdot \langle I\rangle -2\langle R\cdot I\rangle \cdot \langle R\rangle +2\langle R\rangle^{2}\cdot \langle I\rangle \\ \kappa (S,S,S) & =\langle S^{3}\rangle -3\langle S^{2}\rangle \cdot \langle S\rangle +2\langle S\rangle^{3}\\ \kappa (I,I,I) & =\langle I^{3}\rangle -3\langle I^{2}\rangle \cdot \langle I\rangle +2\langle I\rangle^{3}\\ \kappa (R,R,R) & =\langle R^{3}\rangle -3\langle R^{2}\rangle \cdot \langle R\rangle +2\langle R\rangle^{3}. \end{array}$$

The dynamical equations together with the closure relationships (null third-order cumulants) lead to a closed system of ordinary differential equations for nine variables ( ⟨S⟩ , ⟨I⟩ , ⟨R⟩ , $\left\langle S^{2}\right\rangle$ , $\left\langle I^{2}\right\rangle$ , $\left\langle R^{2}\right\rangle$ , ⟨S⋅I⟩ , ⟨S⋅R⟩ and ⟨R⋅I⟩ ).

### Deterministic and stochastic correlations between species

The deterministic approach implies that correlations can be factorized, i.e. ⟨XY⟩=⟨X⟩⟨Y⟩ . Thus, $\langle SI\rangle =\theta \left(K-K_{c}\right)K_{c}\frac{b+\delta }{b+\gamma +\delta }\left(K-K_{c}\right)$ , $\langle SR\rangle =\theta \left(K-K_{c}\right)K_{c}\frac{\gamma }{b+\gamma +\delta }\left(K-K_{c}\right)$ , and $\langle IR\rangle =\theta \left(K-K_{c}\right)K_{c}\frac{\gamma \left(b+\delta \right)}{b+\gamma +\delta }{\left(K-K_{c}\right)}^{2}$ . Consequently, the transition towards infection is determined by non-null values of the correlations terms. Figure 6 shows the comparison between deterministic and stochastic results for ⟨SI⟩ , ⟨SR⟩ and ⟨RI⟩ . In agreement with the results shown in figure 3, the fluctuations acts as a barrier against infection with respect to the deterministic model. Figure 6 also highlights the agreement between stochastic simulations and the MHB analytical calculations.

### Linear stability analysis of the deterministic model: characteristic decay time of infection

The steady-state solution that describes that infection-free system of equations (2.7)–(2.9) is 𝔹=(S=K,I=0,R=0) . The Jacobian matrix that describes the behaviour of small perturbations around that steady-state reads (see [13,14] for details)

$${𝕁|}_{\left(K,0,0\right)}=\left(\begin{array}{ccc} \mu -b & \mu -\beta K & \mu +\delta \\ 0 & -b-\gamma +\beta K & 0\\ 0 & \gamma & -b-\delta \end{array}\right)$$

that leads to the following eigenvalues: $\lambda_{1}=\mu -b$ , $\lambda_{2}=-\left(b+\delta \right)$ and $\lambda_{3}=-\left(b+\gamma \right)+\beta K=\beta \left(K-K_{c}\right)$ . Thus, the stability of the infection-free system is determined by $\lambda_{3}$ : if $K<K_{c}$ then $\lambda_{3}<0$ ( ℕ is stable) and if $K>K_{c}$ then $\lambda_{3}>0$ ( ℕ is unstable). Thus, given an initial condition $I\left(t=0\right)=I^{0}$ , we expect that if $K<K_{c}$ the infected population will decay as $I\left(t\right)≃I^{0}e^{-t\cdot \beta \left(K_{c}-K\right)}$ and, consequently, the probability of observing infected bats at time T can be estimated as $P_{\text{Inf.}}≃I\left(T\right)/I^{0}=e^{-t\beta \left(K_{c}-K\right)}$ . On the other hand, if $K>K_{c}$ the infection is sustained in the deterministic system, i.e. $P_{\text{Inf.}}=1$ . Altogether, the deterministic estimation of the probability of sustained infections reads

$$P_{\text{Inf.}}≃\theta \left(K_{c}-K\right)+\theta \left(K-K_{c}\right)e^{-T\cdot \beta \left(K_{c}-K\right)}.$$
